# Supplementary material for: Reduced SK channel control of mesolimbic dopamine neuron firing drives reward seeking adaptations in chronic pain
Source: bioRxiv. 2025 Oct 7:2025.10.06.680596. Preprint. [Version 1] doi: 10.1101/2025.10.06.680596 (PMC12632544; doi:10.1101/2025.10.06.680596)

Supplemental Figure 1. Acute pain and acute analgesia do not induce behavioral adaptations in progressive ratio responding.

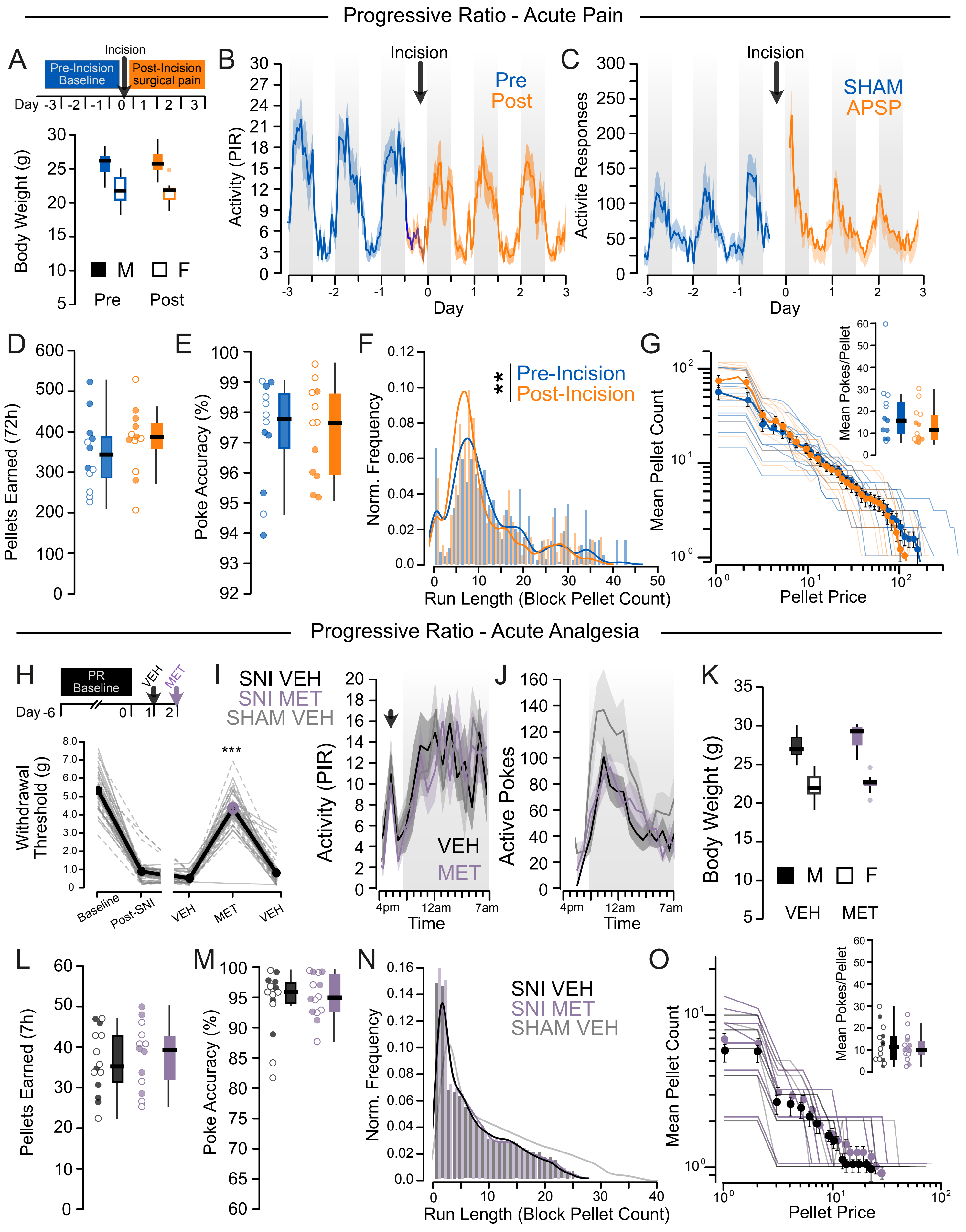

Supplemental Figure 2 SNI-associated behavioral adaptations in reversal learning and progressive ratio task are emulated by systemic haloperidol.

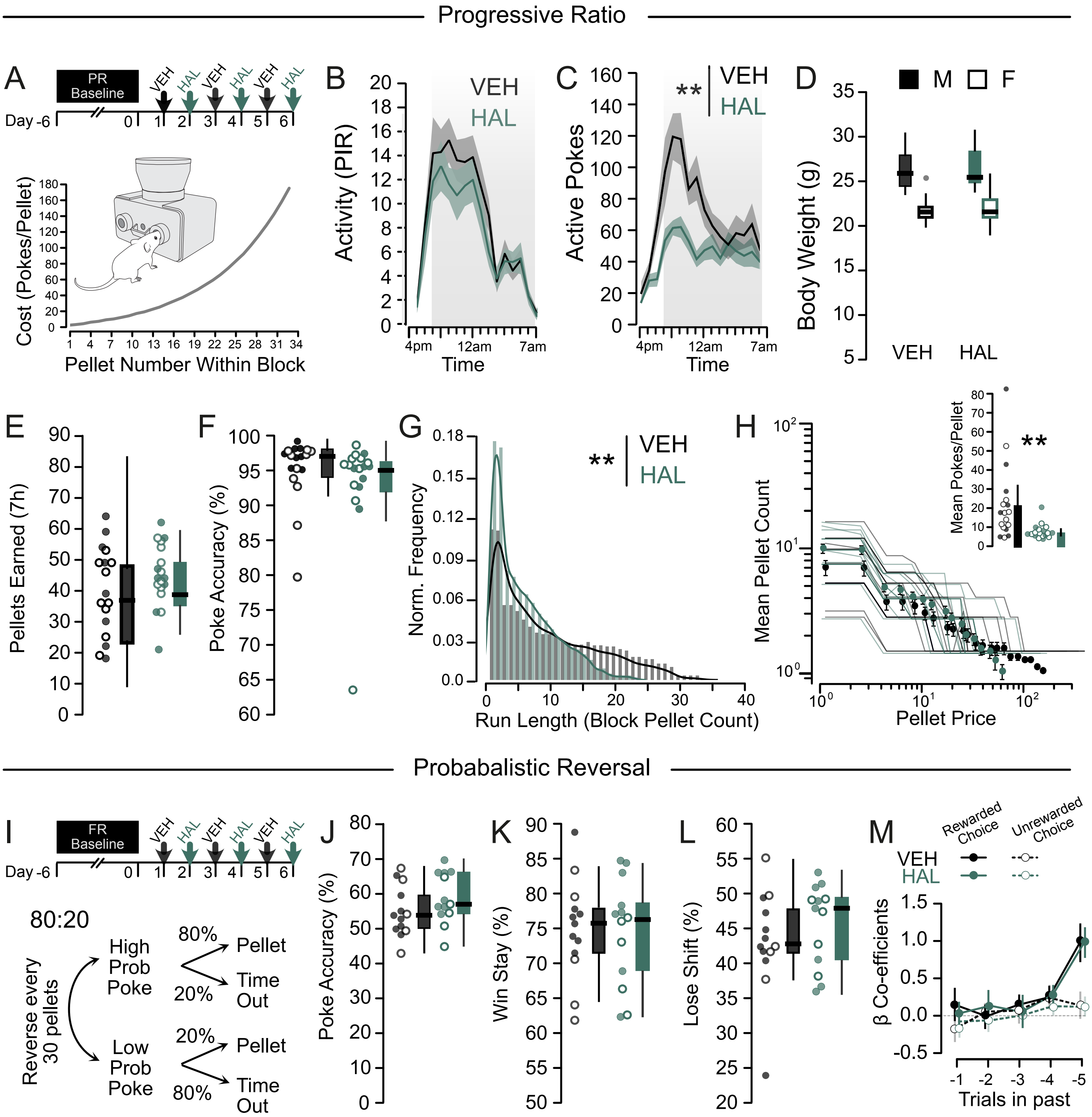

Supplemental Figure 3. Intrinsic membrane properties following SNI.

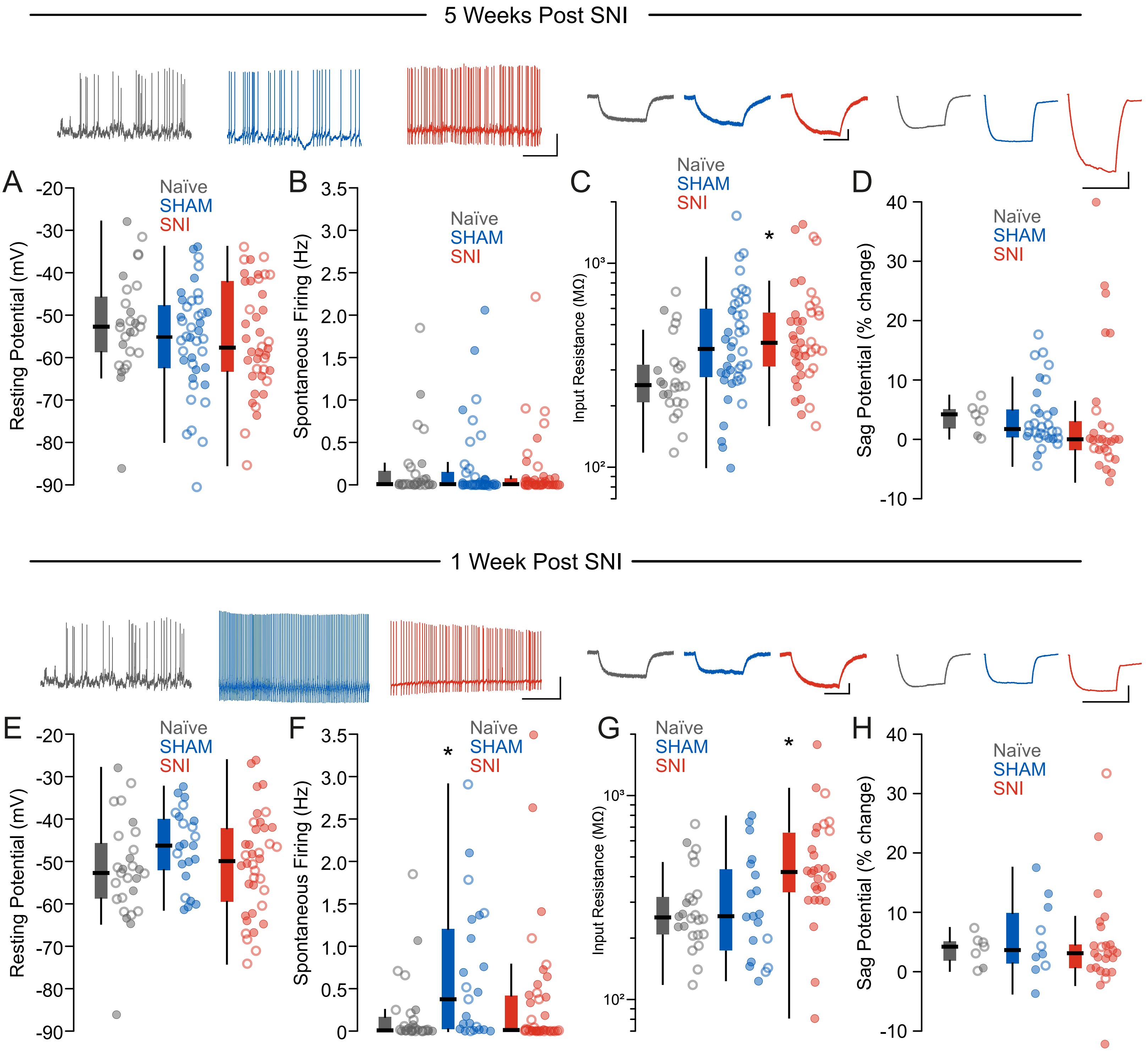

Supplemental Figure 4. Changes in A-type potassium currents do not account for biophysical changes in mesolimbic DA neurons or behavior following SNI.

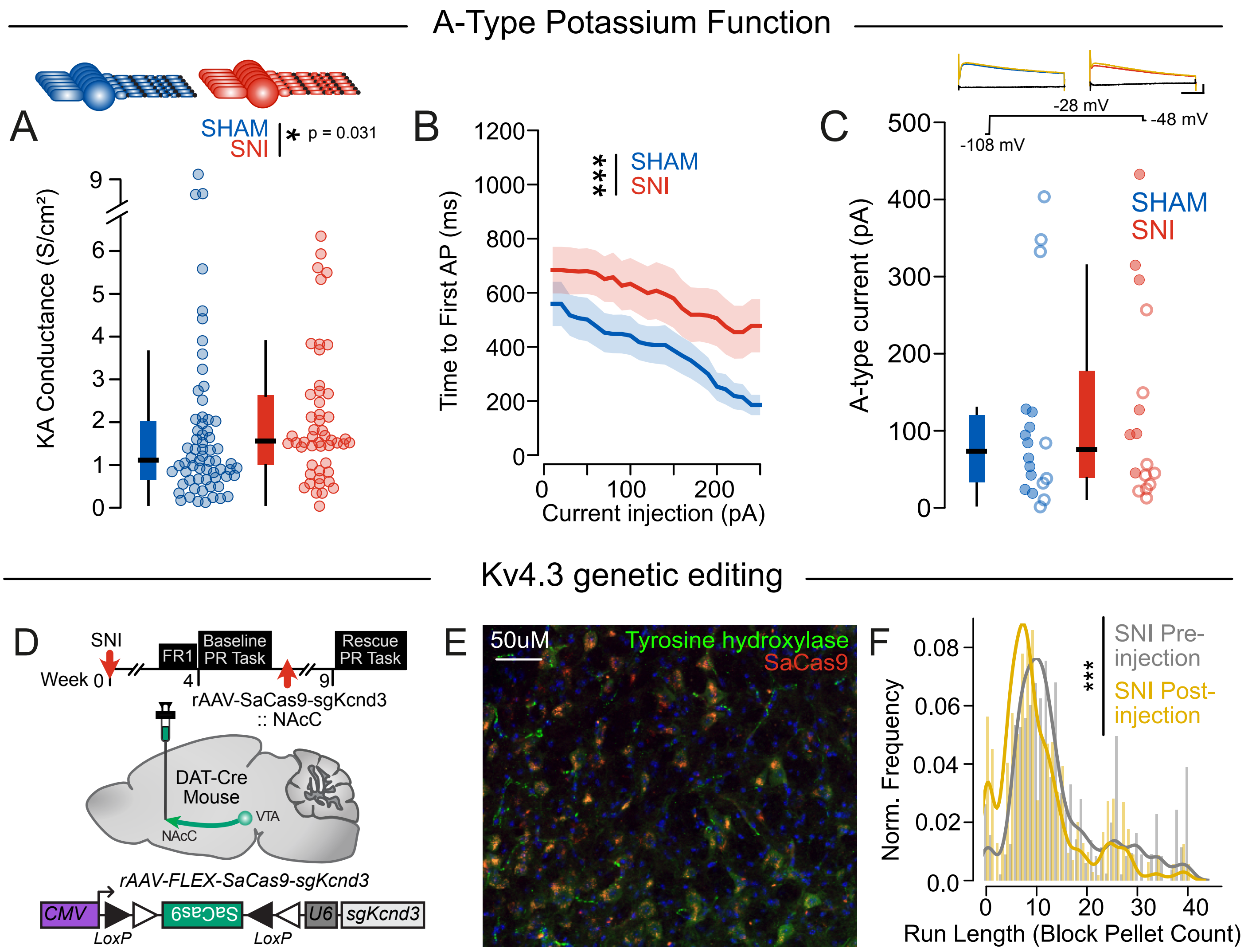

Supplemental Figure 5. Validation of SK overexpression on mesolimbic dopamine neurons and locomotor behavior.

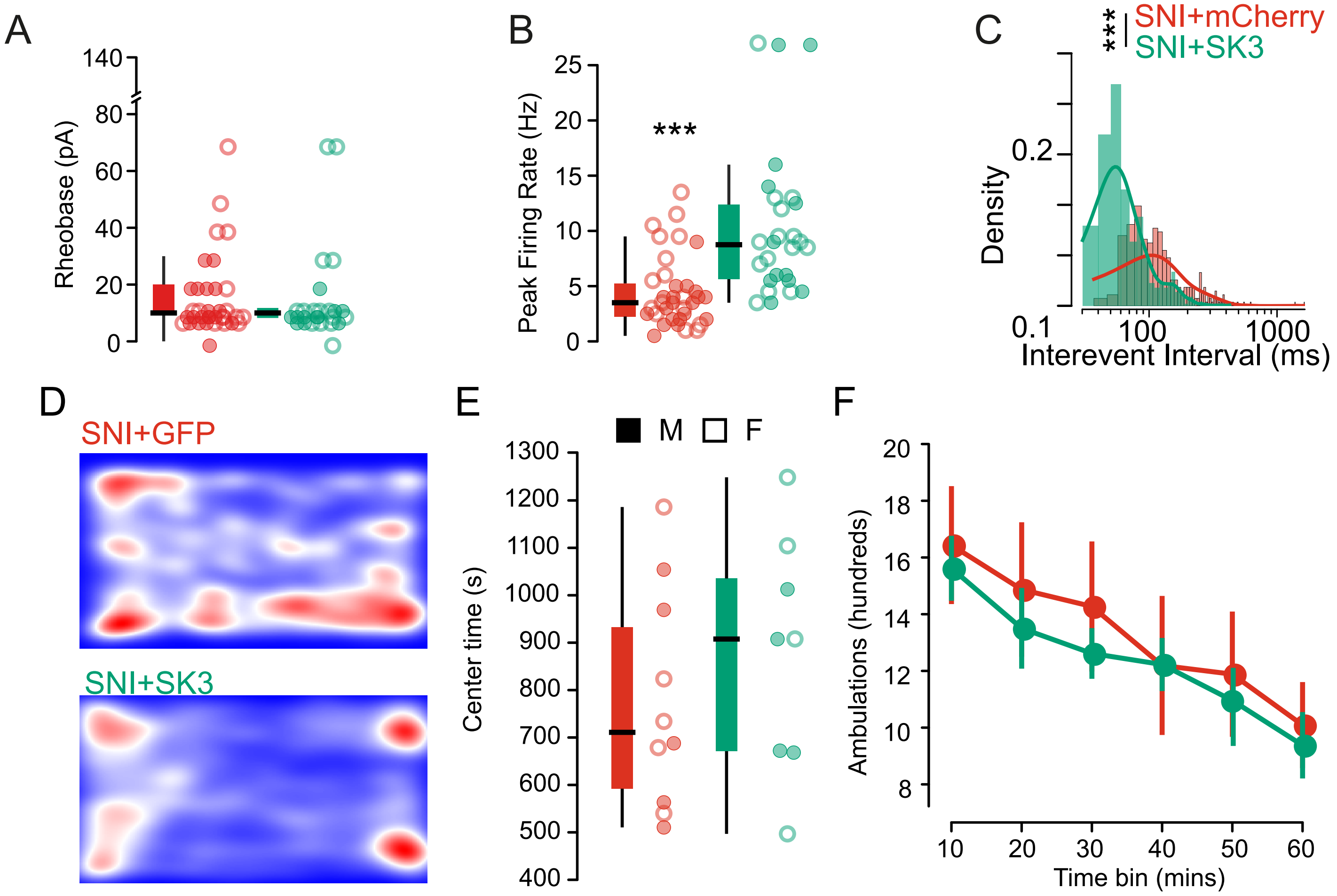

Supplement: Supplement 1 — Supplemental Figure 1. Acute pain and analgesia do not affect strategy on the progressive ratio task. (A) Schematic of acute incisional pain experiment; body weights were not different pre and post paw incision (Pre = 23.04 ± 0.919 sec, Post = 22.56 ± 0.904, F = 0.134, p = 0.717, n=6M/6F). (B) There was a significant effect of time on homecage activity levels, with slight reductions in overall activity levels following incisional pain (F = 7.01, p < 0.001). (C) Mice exhibited significantly greater numbers of active FED responses immediately following paw incision (F = 1.57, p = 0.002). (D-E) There was no difference in pellets earned (FGroup=1.11, p=0.305, Pre = 378.58 ± 24.11, Post = 345.5 ± 25.99) or poke accuracy (FGroup=0.01, p=0.948, Pre = 97.3 ± 4.90 sec, Post = 97.4 ± 4.34) before and after paw incision. (F-G) There was a shift in the distribution of run lengths towards intermediate run lengths (KS: 0.128, p = 0.002), with no differences in demand curves or pokes per pellet before and after acute incisional pain (FGroup=1.89, p=0.184, Pre = 19.47 ± 4.396, Post = 13.56 ± 2.371). (H) Experimental design; MET and VEH injections were administered at 6 pm, prior to onset of progressive ratio testing; there was a significant increase in the withdrawal ratio after metformin injection compared to vehicle injection (F = 87.10, p < 0.0001, n= 6M/8F mice). (I-J) MET had no significant effect on total ambulatory activity (FGroup = 0.308, p = 0.579) or on active FED responses (FGroup = 0.858, p = 0.357); mean of contemporaneous SHAMs are indicated in grey. (K) There were no differences in body weight between VEH and MET conditions (FGroup=0.75, p=0.390, VEH = 24.40 ± 0.62 g, MET = 25.18 ± 0.66). (L-M) MET did not affect the number of pellets earned (FGroup=0.25, p=0.620, VEH = 38.14 ± 2.035, MET = 36.64 ± 2.085) or response accuracy (FGroup=0.37, p=0.549, VEH = 94.19 ± 0.94, MET = 95.26 ± 1.427). (N-O) Relative to VEH, mice treated with MET did not exhibit a shift in distr [file media-1.pdf]
